# The impact of the glucagon‐like peptide 1 receptor agonist liraglutide on the streptozotocin‐induced diabetic mouse kidney proteome

**DOI:** 10.14814/phy2.13994

**Published:** 2019-02-25

**Authors:** Leena Liljedahl, Maiken H. Pedersen, James N. McGuire, Peter James

**Affiliations:** ^1^ Department of Immunotechnology Lund University Lund Sweden; ^2^ Novo Nordisk A/S Måløv Denmark

**Keywords:** Diabetic kidney damage, GLP‐1R agonist, kidney proteome, liraglutide

## Abstract

In diabetes mellitus (DM), the kidneys are exposed to increased levels of hyperglycemia‐induced oxidative stress. Elevated amounts of reactive oxygen species (ROS) are believed to provoke ultrastructural changes in kidney tissue and can eventually result in DM late complications such as diabetic nephropathy. While it is reported that glucagon‐like peptide 1 receptors (GLP‐1R) are present in the kidney vasculature, the effects of GLP‐1 on the kidney proteome in DM is not well described. Thus, we set out to investigate potential effects on the proteomic level. Here the effects of GLP‐1R agonism using the GLP‐1 analogue liraglutide are studied in the kidneys of streptozotocin (STZ)‐treated mice (*n *= 6/group) by label‐free shotgun mass spectrometry (MS) and targeted MS. Unsupervised and supervised multivariate analyses are followed by one‐way ANOVA. Shotgun MS data of vehicle and liraglutide‐treated mouse groups are separated in the supervised multivariate analysis and separation is also achieved in the subsequent unsupervised multivariate analysis using targeted MS data. The mouse group receiving the GLP‐1R agonist liraglutide has increased protein abundances of glutathione peroxidase‐3 (GPX3) and catalase (CATA) while the abundances of neuroplastin (NPTN) and bifunctional glutamate/proline–tRNA ligase (SYEP) are decreased compared to the STZ vehicle mice. The data suggest that GLP‐1R agonism mainly influences abundances of structurally involved proteins and proteins involved in oxidative stress responses in the STZ mouse kidney. The changes could be direct effects of GLP‐1R agonism in the kidneys or indirectly caused by a systemic response to GLP‐1R activation.

## Introduction

Worldwide, the diabetes mellitus (DM) late complication diabetic nephropathy (DN) is a substantial cause of chronic kidney disease. Diabetic kidney disease is characterized by increased leakage of serum albumin into the urine, decreased glomerular filtration rate and structural lesions. Increased hyperglycemia‐induced oxidative stress, from time to time present in DM regardless of DM subtype, is reported to be mediated by mitochondrial overproduction of reactive oxygen species (ROS; Giacco and Brownlee 2010). This excess of ROS could have harmful effects on protein and tissue structure (Davies 1987) and participate in the early ultrastructural changes taking place in DN (Ota et al. 1993; Tervaert et al. 2010a). Another key mechanism in the development of DN is the hyperglycemia‐induced increase of advanced glycation end products, also inducing increased amounts of ROS and cross‐linking of the extracellular matrix (ECM) (Tan et al. 2007). Over time, irreversible morphological damage develops in DN with basement membrane thickening, mesangial expansion, and interstitial fibrosis (Brito et al. 1998; Tervaert et al. 2010b; Forbes and Cooper 2013).

Approximately 40% of patients with type 1 DM have metabolic syndrome and the prevalence is increased with advanced DN and poor glycemic control (Thorn et al. 2005). In obese patients with type 1 DM, the glucagon‐like peptide 1 receptor (GLP‐1R) agonist liraglutide as additional treatment to insulin was shown to improve glycemic control and reduce body weight (Kuhadiya et al. 2013a). However, a second study showed that not all overweight and obese type 1 DM patients benefitted from the additional treatment with liraglutide (Kuhadiya et al. 2016). A recent 1 year randomized clinical study thus concluded that the addition of liraglutide to insulin treatment of type 1 diabetes had beneficial effects on parameters related to diabetes although no difference in renal outcomes were reported (Dandona et al. 2018). In type 2 DM, a clinical study reports on fewer renal outcomes in the group receiving liraglutide compared to the placebo group. Liraglutide mainly reduced the prevalence of persistent macroalbuminuria (Mann et al. 2017).

In the kidney, the GLP‐1R has been detected in smooth muscle cells in the vascular walls of arteries and arterioles in primates (Pyke et al. 2014) and rodents (Jensen et al. 2015). In a Japanese study, it was reported that serum dipeptidyl peptidase‐4 (DPP‐4) activity was increased in young‐adult type‐1 DM, suggesting a pathophysiological influence of DPP‐4 in type 1 DM (Osawa et al. 2016). One function of active DPP‐4 is that it inactivates incretins like GLP‐1.

In the kidneys of streptozotocin (STZ)‐induced diabetic rats, the GLP‐1R agonist liraglutide was previously observed to decrease oxidative stress markers (Hendarto et al. 2012). We hypothesized that GLP‐1R agonism could have an effect on protein levels in the STZ‐induced diabetic mouse kidney, either directly by acting on GLP‐1Rs in the kidney or through systemic effects by GLP‐1R activation. Hence, to enhance the understanding of the molecular effects of systemic GLP‐1R agonism on the kidneys in early diabetic kidney disease, we investigated the effects of the GLP‐1R agonist liraglutide on the STZ mouse model. This experimental mouse model is hyperglycemic and has a reduced *β*‐cell mass, and early stages of diabetic kidney disease with increased albumin excretion rate (AER) (Breyer et al. 2005). General traits in the glomeruli from STZ‐induced diabetic rats include decreased capacity of oxidative stress detoxification, increased cell differentiation and possibly renal matrix expansion compared to healthy rats (Kim et al. 2014). By combining discovery‐based label‐free shotgun mass spectrometry (MS) and multivariate analysis, our aim was to dig deeper into the overall effect of GLP‐1R agonism on the kidney proteome in STZ‐induced diabetic mice. We have previously conducted similar research on the obese db/db mouse model (Liljedahl et al. 2017).

## Materials and Methods

### Animals

All handling and use of animals in the present study was approved by The Danish Animal Experiments Inspectorate and were carried out according to the guidelines of “The Council of Europe Convention for the Protection of Vertebrate Animals used for Experimental and other Scientific purposes.”

Male 129SV mice were purchased from Charles River, Germany, at 6 weeks of age. The mice were housed in groups of 10/cage in the animal unit facility at Novo Nordisk, Denmark under controlled temperature (19–21°C) with a 12–12 h light‐dark cycle with lights on at 6 AM. Altromin 1324 food pellets and tap water was served ad libitum. After 2 weeks of acclimatization, the mice were randomized into two groups, whereof one group was given two freshly dissolved STZ injections intraperitoneally at a dose of 125 mg/kg with 3 days between doses. Two weeks post‐STZ treatment, the STZ mice were randomized into two dosing groups, vehicle control and liraglutide. Blood glucose (BG) measurements were conducted and mice showing elevated BG above 16 mmol/L were included in the experiment. Liraglutide (Novo Nordisk, Maaloev, Denmark) was administered subcutaneously (s.c.) once daily and the dose was escalated over 3 days with a dose of 0.33 mg/kg on day 1, 0.66 mg/kg on day 2, and the final dose of 1 mg/kg from day 3 and the following 8 weeks until termination. The dose escalation serves to prevent acute nausea, which is a common temporary side effect of liraglutide treatment. One group of mice were not injected with STZ and served as healthy controls. These and the diabetic control group were injected with vehicle when liraglutide treatment started. Vehicle dosing was done s.c. with 4 mL/kg QD with the vehicle composition: pH 7.4; 20 mmol/L phosphate; 130 mmol/L sodium chloride; 0.05% polysorbate 80.

### In vivo measurements

Glycated hemoglobin A_1c_ (HbA_1c_(%)) was analyzed at 6 and 10 weeks after STZ dosing on a Cobas 6000 autoanalyzer (Roche Diagnostics Ltd, Rotkreuz, Switzerland), BG was analyzed weekly on a Biosen S‐line/5040 (EKF‐diagnostics, Magdeburg, Germany) and urine AER was analyzed at 2, 6 and 10 weeks after STZ dosing. Urine was collected individually during 24 h of metabolic caging (Techniplast S.p.A., Buguggiate, Italy) and AER was determined using a sandwich ELISA (Bethyl Labs, Montgomery, TX). Body weight, kidney weight per body weight (%), and AER normalized to body weight (g) were calculated. The animals were killed 10 weeks after the STZ intervention by perfusion under isoflurane anesthesia with 20 mL 0.9% NaCl with heparin (10 IU/mL). Kidneys were weighed individually after having the surrounding fat removed and were snap frozen in liquid nitrogen.

**Figure 1:**
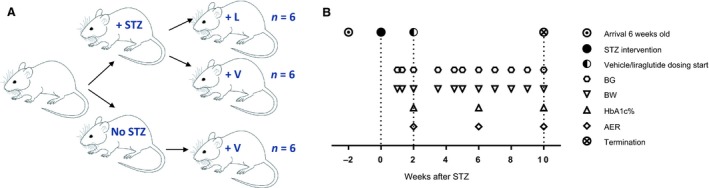
Overview of study. (A) Male Charles River mice were divided into healthy control receiving vehicle (+V), *n *= 6, streptozotocin (STZ) vehicle (+V), *n *= 6 and STZ liraglutide (+L), *n *= 6. (B) Timeline of timepoints when the mice had their blood glucose (BG), body weight (BW), albumin excretion rate (AER), and HbA1c% measured.

A total of 18 mice were included in the study, *n *= 6 healthy control vehicle‐dosed mice, *n *= 6 STZ vehicle‐dosed mice, and *n *= 6 STZ and liraglutide‐dosed mice. An overview of the study is shown in Figure 1.

### Protein purification and sample preparation

Solvent percentages are reported as (v/v). Acetonitrile (ACN), formic acid (FA) 2,2,2‐trifluoroethanol (TFE), ammonium acetate, ammonium bicarbonate, phosphate buffered saline (PBS), sodium chloride, sodium sulfite, N‐acetyl‐cysteine, iodoacetamide (IAA) and dithiothreitol (DTT) were purchased from Sigma‐Aldrich (Stockholm, Sweden). From a larger section of snap frozen tissue from the right kidney, consisting of a mixture of cortex and medulla, 50 mg tissue was transferred to a Denator Stabilizor T1 (Svensson et al. 2009) (Denator, Gothenburg, Sweden) for termination of enzymatic processes. Thereafter, tissue homogenization was done using a bead beater with Zirconia beads (BioSpec Products, Inc., Bartlesville, OK) for 4 times 1 min and kept on ice in between. Samples were lysed in ice cold 0.5 mL 50% TFE/50% PBS for 10 min, 100 rpm shaking at 4°C, then 250 *μ*L 100 mmol/L ammonium bicarbonate pH 8 was added and the samples were centrifuged at 4°C and 2000*g* for 30 min. The supernatant was incubated shaking at 400 rpm for 2 h at 60°C, then samples were centrifuged for 10 min at room temperature (RT) and 2000*g*. Thereafter, the supernatant was reduced for 30 min with 5 mmol/L DTT at 60°C, 800 rpm, alkylated for 30 min with 25 mmol/L IAA at RT in the dark followed by quenching for 15 min with 30 mmol/L *N*‐acetyl‐cysteine at RT in the dark, all at pH 8. Digestion of samples was done with 20 *μ*g sequencing grade modified trypsin (Promega, Madison, WI) per sample at 1000 rpm and 37°C overnight. Reverse‐phase (500 mg) C_18_ chromatography (Waters, Milford, MA) was used for cleanup of the digest and peptides were eluted with 0.1% FA/50% ACN and then reduced on a SpeedVac (ThermoScientific, Waltham, MA) to 100 *μ*L final volume. In order to reduce the complexity of the samples before MS analysis, all samples were separated into two fractions per sample; one fraction containing glycosylated peptides (investigated in a separate study [Liljedahl et al. 2016]) and the nonglycosylated fraction studied here. The complete sample was oxidized in 20 mmol/L sodium acetate/100 mmol/L sodium chloride at pH 5 with sodium meta‐periodate (Pierce, SDS diagnostics, Falkenberg, Sweden) added to 8 mmol/L final concentration and incubated at 6°C at 600 rpm in the dark for 60 min. After termination with 30 mmol/L sodium sulfite in the dark for 15 min at RT, the samples were coupled at RT overnight to 0.5 mL hydrazide Affi‐gel (HZ) (BioRad, Hercules, CA) in a 50% slurry of 100 mmol/L sodium acetate/1 mol/L sodium chloride at pH 4.5 and 37°C with gentle vertical agitation. The supernatant (nonglycosylated peptide fraction) was collected and combined with the first wash of the HZ resin with 80% ACN, thereafter reduced on a SpeedVac, cleaned up by reverse‐phase C_18_ chromatography (100 mg, Waters) and dried on the SpeedVac.

### MS analyses

The dried samples were dissolved in 5% ACN and 0.1% FA. Mobile phase A was water/0.1% FA, mobile phase B ACN/0.1% FA. Shotgun analysis was done on a linear trap quadrupole (LTQ) Orbitrap XL mass spectrometer (Thermo Electron, Bremen, Germany). All 18 samples were run once. Sample loading was done at a constant flowrate of 10 *μ*L/min onto the precolumn (C18 PepMap100, 5 *μ*m, 5 mm × 0.3 mm, LC Packings, Amsterdam, Netherlands) and then samples were separated on a 10 *μ*m fused silica emitter, 75 *μ*m × 15 cm (PicoTip™ Emitter, New Objective Inc., Woburn, MA), which was packed in‐house with Reprosil‐Pur C18‐AQ resin (3 *μ*m Dr. Maisch GmbH, Ammerbuch‐Entringen, Germany). Peptides were eluted from the analytical column using a linear gradient of mobile phase B developed from 3% to 35% during 90 min for label‐free quantification. The gradient was followed by 20 min column washing with 90% B and a 15 min reequilibration with 3% B. Peptide analysis was done using data‐dependent acquisition as described in Liljedahl et al. 2016. Data were acquired using the Xcalibur software, version 2.0.7 (Thermo Fischer Scientific, Hägersten, Sweden). The shotgun MS data have been deposited to the ProteomeXchange Consortium (Vizcaíno et al. 2014) via the PRIDE partner repository (Vizcaíno et al. 2013) with the dataset identifier PXD003007 and 10.6019/PXD003007. Selected reaction monitoring (SRM) LC MS/MS analysis was done on an Eksigent 2D NanoLC system (Eksigent technologies) interfaced with a triple stage quadrupole (TSQ) Vantage mass spectrometer (Thermo Scientific, San Jose, CA). The separation system setup was as described above for the shotgun analysis. The LC gradient, TSQ operating mode and data acquisition were as described previously (Liljedahl et al. 2017). Three of the samples were run in triplicate throughout the series to calculate %CV for normalized values. The three samples run in triplicate revealed a maximum CV of 6% and an average below 1% of the normalized intensities. The SRM MS data are available through PASSEL (Farrah et al. 2012) via Peptide Atlas with the dataset identifier PASS00839.

### Data analyses

Shotgun data raw files were imported to Progenesis QI v.1.0.5156.29278 (Nonlinear Dynamics, Waters), coupled to the Mascot version 2.4 search engine (MatrixScience, London, UK) and processed data were searched against UniprotKB mouse 2013.05 with reversed sequences and settings as previously described (Liljedahl et al. 2017). The 7167 identified peptides were assembled into 1203 proteins and quantification was done based on nonconflicting peptide intensities (peptide data are available in Table S1). Both peptide and protein raw data were exported for subsequent statistical analysis. In addition, shotgun raw data files were converted into mzData (XML), submitted to Proteios version 2.17.0 (Häkkinen et al. 2009), where a spectral library for SRM assays were constructed using Mascot search parameters as described (Liljedahl et al. 2017). For both Progenesis and Proteios, peptides and proteins were filtered at 5% false discovery rate.

Confirmation of the relative protein levels detected in the shotgun data was done by targeted SRM MS of proteotypic peptides (Aebersold et al. 2013). Proteins were selected for SRM based on the multivariate analysis of the shotgun data and peptides were checked against an in silico digestion of the complete Uniprot KB mouse proteome 2014.12.18. Synthetic peptides were from JPT (Berlin, Germany). Shotgun expression signatures from proteins and the corresponding peptides to be used in the SRM assay were inspected. Here, 106 peptides with a minimum of four transitions per peptide from 71 proteins were examined in the SRM assay using the SRM software Skyline v. 2.6.0.7176 (MacLean et al. 2010) (MacCoss lab, Washington) where the SRM method was constructed and the obtained SRM raw data were manually inspected. The SRM assay with all transitions is shown in Table S2. One peptide was removed due to poor transition spectra. Peak areas for all transitions of a peptide were integrated and added together to obtain the total peak area for each peptide. Technical replicates were merged for all further analyses and the data were imported to the software Normalyzer 1.1.1 (Chawade et al. 2014) in order to assess the ideal normalization method. After thorough comparison of Loess‐R (local normalization) and Loess‐G (global normalization), Loess‐G was selected for this dataset (Smyth 2005). Nine peptides from eight proteins were included in the SRM assay as controls for normalization based on their stable expression detected in the shotgun dataset and all nine peptides remained unchanged in the SRM dataset (Table S3). Shotgun MS is a biased method in the sense that the detection of a peptide is dependent on its ability to ionize. Some proteins with too few identified peptides in the shotgun data were not further studied in the SRM MS analysis in this study, although others previously have shown altered protein abundances in rodent models of diabetic kidney disease. One example of such a protein is angiotensin‐converting enzyme 1 (ACE) (Anderson et al. 1993; Wysocki et al. 2006).

### Multivariate analyses

Principal component analysis (PCA) (Ringnér 2008) and orthogonal projection to latent structures discriminant analysis (OPLS‐DA) were used for multivariate analyses to reduce the dimensionality of the dataset (SIMCA v. 15, Umetrics, Umeå, Sweden) (Bylesjö et al. 2006; Wiklund et al. 2008). The SIMCA *Shared and Unique Structure* (SUS) plot and *Variable Importance for the Projection* (VIP) plots were used as in Liljedahl et al. 2017.

When the animals were killed, the degree of perfusion was not equivalent in all of them, which could be seen in the PCA in SIMCA when comparing sample and variable (protein) distribution. Albumin, *β*‐globin subunits, and hemoglobin A1 and A2 were identified as variables depending on the degree of perfusion, and in total 12 blood proteins were excluded from all further analyses from all samples.

In the OPLS‐DA model, the three groups of mice were defined as discriminants. The statistical parameters R2X(cum), R2Y(cum), and Q2(cum), describing the fit of each model, is shown in Table S4. The use of the SUS‐plot in the data interpretation of the OPLS‐DA model enabled the protein expression of all three mice groups to be compared in the same plot. First the healthy control versus STZ vehicle and the STZ liraglutide versus the STZ vehicle mouse groups were compared in S‐plots, then the two comparisons were combined in the SUS plot with the STZ vehicle group as a common reference. The goal of the analysis was to identify proteins that had significantly different protein levels affected firstly by the STZ intervention and secondly by the liraglutide administration. In the S‐plots and SUS‐plot, all variables with a value above 1 in the VIP plots were examined.

For the proteins in the SRM assay with a significant difference between any mouse group, the normalized data were imported into a separate project in SIMCA. PCA was performed to validate whether the three groups of mice were separated only based on those variable values.

### Univariate statistical analyses

Here, BG, HbA_1c_(%), AER, kidney weight divided by body weight (KW/BW) and protein data in the text are presented with mean ± standard deviation (SD). Mouse parameters and protein data in graphs are presented with mean and standard error of mean (SEM) and 95% confidence interval (CI), respectively. One‐way ANOVA with Tukey post hoc analysis was done for statistical comparisons in GraphPad Prism 6 (La Jolla, CA) or AmberBio (Amber BioScience, Lund, Sweden). Brown–Forsythe′s test was calculated simultaneously in Prism to assess differences in the intragroup variance and if found significant (which it was for uromodulin, aquaporin‐2, tinag, and annexin‐5) the proteins were not further analyzed. Consequently *P *< 0.05 was considered significant in all tests.

## Results

The STZ‐induced diabetic mice were divided into two groups dosed with liraglutide (STZ liraglutide, *n *= 6) or vehicle (STZ vehicle, *n *= 6). A group of healthy mice dosed with vehicle, *n *= 6 served as a healthy control (Fig. 1).

### Investigated mouse parameters

**Figure 2:**
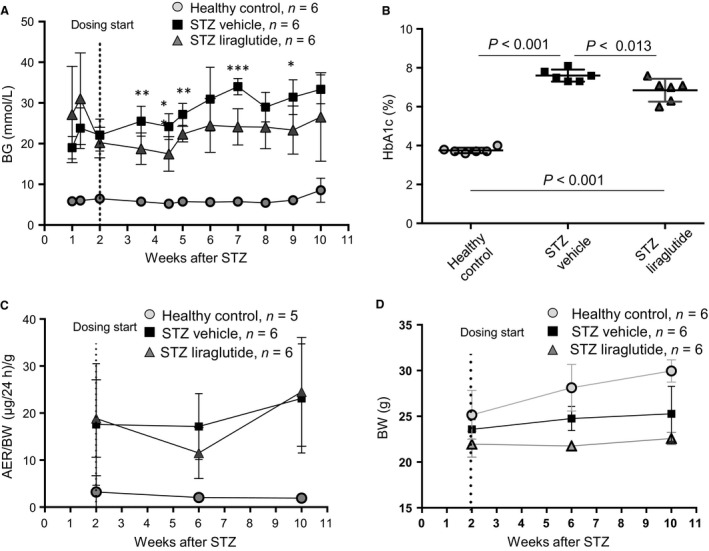
The effect of liraglutide on mouse parameters in the STZ model. (A) Mean blood glucose (BG) was at all measured time‐points lower in the STZ liraglutide group as compared to the STZ vehicle group. Significant differences at the measuring time‐points between the STZ vehicle and STZ liraglutide groups are indicated with asterisks, *P < 0.05, **P < 0.01 and ***P < 0.001. (B) Liraglutide decreased HbA_1c_(%) compared to the STZ vehicle group, measured after 8 weeks of dosing. (C) Albumin Excretion Rate (normalized to gram (g) of body weight) was increased in the two STZ mouse groups compared to the healthy control group at all measured time points. (D) Body weight (BW). Data are presented as mean with SD and statistical analysis is made using one‐way ANOVA with Tukey post hoc test to correct for multiple comparisons, *P *< 0.05 is considered significant.

BG and HbA_1c_(%) levels were significantly higher in the two STZ mouse groups compared to the healthy control mice at all measuring occasions (illustrated in Fig. 2A–B and data shown in Table S1). The healthy control mice had stable BG levels between 5.2 ± 0.5 and 8.6 ± 3.0 mmol/L (mean ± SD) at all measured time points and the terminal HbA_1c_(%) was 3.8 ± 0.1%. In the STZ vehicle group, terminal HbA_1c_(%) was 7.6 ± 0.3% and in the STZ liraglutide group terminal HbA_1c_(%) was 6.9 ± 0.6%, which was significantly decrease compared to the STZ vehicle group (*P *< 0.013, Fig. 2B). There was no significant difference between the terminal BG levels in the STZ vehicle and STZ liraglutide groups, probably due to high BG levels in one animal in the STZ liraglutide group at 48 mmol/L. The terminal mean BG levels were 33 ± 4 mmol/L in the STZ vehicle group and 26 ± 11 mmol/L in the STZ liraglutide group. After dosing start, the mean BG value in the STZ liraglutide group was consistently lower than the mean BG value in the STZ vehicle group with significant differences between the two STZ groups at five out of eight measuring time points and a trend toward a difference at one additional time point. The AER was analyzed at three time points (shown normalized to body weight in grams [g] in Fig. 2C, raw data is shown in Fig. S1A). At baseline and at the termination of the study, the mean AER was significantly increased in the STZ groups relative to the healthy control group. The mean AER of the healthy control varied between 55 ± 26 and 75 ± 82 *μ*g/24 h (1.9 ± 0.9 to 3.2 ± 3.5 *μ*g/24 h/g BW), the STZ vehicle mean AER between 408 ± 281 to 578 ± 288 *μ*g/24 h (17.1 ± 7.0 to 23.1 ± 11.6 *μ*g/24 h/g BW) and the STZ liraglutide mean AER between 250 ± 117 (at 4 weeks) and 541 ± 160 *μ*g/24 h (11.5 ± 5.4 at 4 weeks to 24.5 ± 11.5 *μ*g/24 h/g BW). From dosing start to termination of the study, the body weight increased in the healthy control mice with 19% (from 25.2 ± 2.7 to 30.0 ± 1.2 g), in the STZ vehicle group with 7% (from 23.6 ± 1.1 to 25.3 ± 3.0 g) and in the STZ liraglutide group with 3% (from 22.0 ± 1.4 to 22.6 ± 0.7 g) as seen in Figure 2D. At termination of the study, the kidney weight of the right kidney (used in this study) was 0.242 ± 0.012 g in the healthy control group, 0.258 ± 0.017 g in the STZ vehicle group and 0.221 ± 0.014 g in the STZ liraglutide group (Fig. S1B). The proportion of kidney weight per body weight was ~0.8% in the healthy control mice and 1% in the STZ mouse groups (Fig. S1C).

### Effects of liraglutide on the mouse kidney proteome

**Figure 3:**
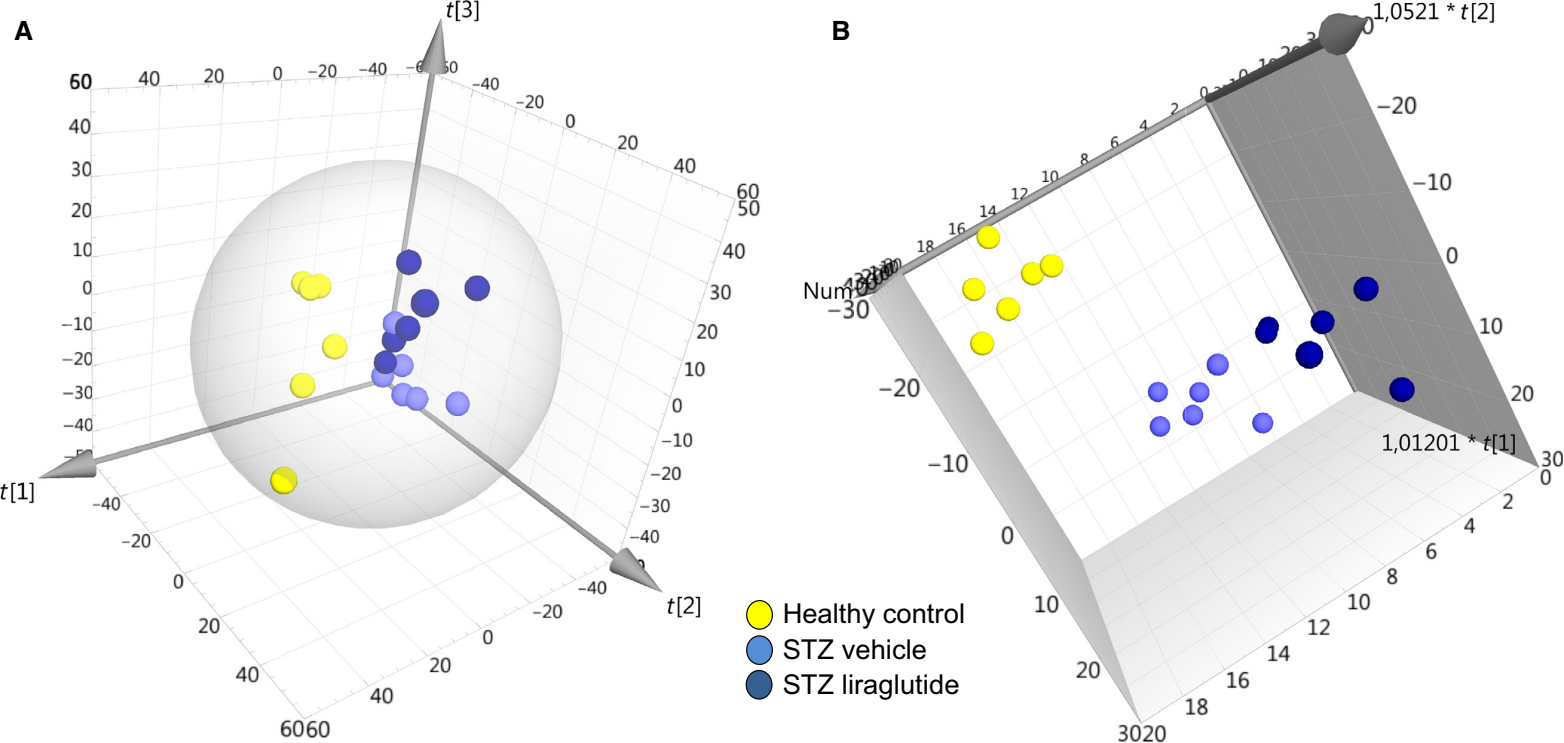
Separation of mouse groups using multivariate analyses. (A) In the unsupervised PCA based on 1191 protein abundances from the shotgun analysis the healthy control group is clearly separated from the two STZ groups, but there is no separation within the two STZ groups. The two first components are R2X[1]* *= 0.223 and R2X[2]* *= 0.194. (B) Separation of the STZ liraglutide and STZ vehicle mouse groups was achieved in the supervised OPLS discriminant analysis.

**Figure 4:**
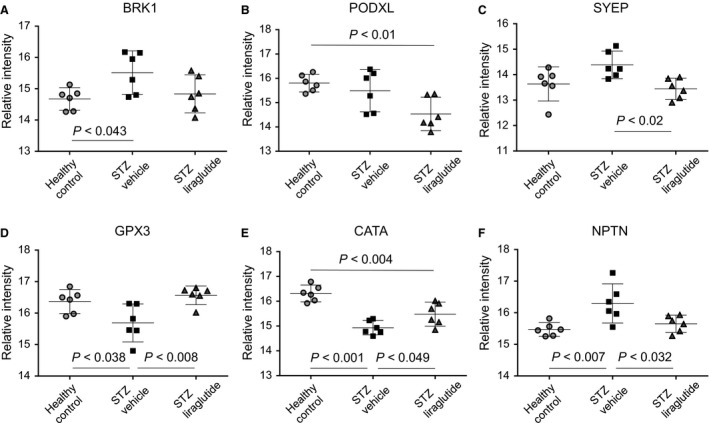
Effect of GLP‐1 receptor agonism on protein abundances. (A) STZ treatment increased the levels of the structurally involved protein BRK1 and B) liraglutide decreased the level of PODXL, in both cases compared to the healthy control mice. C) The level of SYEP was decreased by liraglutide compared to the STZ vehicle mice. D) Increased levels of the antioxidant defence enzymes GPX3 (peptide NSC shown here) and E) CATA, (peptide FNS shown here) were seen compared to the STZ vehicle mice. F) STZ treatment increased the level of NPTN (peptide NGV shown here) and liraglutide decreased it towards the level in the healthy control mice. One‐way ANOVA where mean and 95% confidence interval are indicated, *P*‐values are calculated in GraphPad PRISM using Tukey post hoc test and *P *< 0.05 is considered significant.

Shotgun MS analysis resulted in the identification of 1191 proteins used for unsupervised PCA and supervised OPLS‐DA. The two STZ groups were not separable by PCA, but the healthy control group was clearly defined (Fig. 3A). The three groups were defined as discriminants in the OPLS analysis (Fig. 3B). Here, the STZ vehicle and STZ liraglutide groups were separating, revealing the group‐specific protein expression. Based on their importance for the separation of the three mouse groups in the OPLS‐DA, 63 proteins were selected for quantitative confirmation using SRM MS (Aebersold et al. 2013). The protein response to STZ and liraglutide, compared to the response to STZ alone allowed the placement of the proteins with significant abundance levels into the five sets listed in Table 1 with supplementary data of protein intensities and peptides used for protein identification shown in Tables S5–S6. All shown protein abundances are normalized intensity‐based relative abundances. In the first set, the protein abundances were regulated by the STZ intervention. In the second set significant differences were found between the healthy control and the STZ vehicle mice. The group of mice given liraglutide was not significantly different from either the healthy control or the STZ vehicle mice, indicating a small effect of liraglutide on the protein abundance in the STZ mice toward the level in the healthy control mice. Included in this group was brick‐1 (BRK1) shown in Figure 4A and Figure S2A. In the third set, significant differences in protein abundance were seen between the healthy control mice and the STZ liraglutide mice, including podocalyxin (PODXL) shown in Figure 4B and Figure S2B. In the fourth set, there was a difference between the STZ vehicle and STZ liraglutide mouse groups. Included in this set was bifunctional glutamate/proline‐tRNA ligase (SYEP) shown in Figure 4C and Figure S2C. In the fifth set, the STZ vehicle mice were significantly different from both the healthy control and the STZ liraglutide mice and for the proteins in this set liraglutide reversed the effect of the STZ intervention to varying extents. In this set, the protein levels of glutathione peroxidase‐3 (GPX3) and catalase (CATA) were decreased in the STZ vehicle mice compared to the healthy control mice, and liraglutide normalized the protein abundance levels in the STZ mice as seen in Figures 4D–E and Figure S2D–E. In contrast, the level of neuroplastin (NPTN), shown in Figure 4F and Figure S2F was increased in the STZ vehicle mice compared to the healthy control mice and liraglutide decreased the protein level toward the levels in the healthy control mice.

**Table 1:** Proteins with SRM MS Verified Significant Protein Levels

Uniprot accession	Protein name	Short name	Direction of change
Set 1	Mainly mouse model specific, smaller STZ vehicle‐STZ liraglutide differences		STZ vehicle and STZ liraglutide versus healthy control
O70439	Syntaxin 7	STX7	Down
Q8C165	Carboxypeptidase PM20D1	P20D1	Down
Q62165	Dystroglycan	DAG1	Down
P11352	Glutathione peroxidase 1	GPX1	Up
P14069/Q545I9	Protein S100‐A6	S100A6	Up
P00920	Carbonic anhydrase 2	CAR2	Up
Q8VDN2	Sodium/potassium‐transporting ATPase subunit alpha‐1	AT1A1	Up
Set 2	Vehicle differences only		STZ vehicle versus healthy control
Q9JHJ8	Inducible T‐cell co‐stimulator ligand	ICOSL	Up
Q9WVA2	Mitochondrial import inner membrane translocase subunit Tim8a	TIM8A	Up
Q9WV98	Mitochondrial import inner membrane translocase subunit Tim9	TIM9	Up
Q91VR8	Brick 1	BRK1	Up
Set 3	Liraglutide versus healthy vehicle		STZ liraglutide versus healthy control
Q8QZW3	Family with sequence similarity 151, member A	F151A	Down
P05202	Aspartate aminotransferase	GOT2	Up
Q9R0M4	Podocalyxin	PODXL	Down
Set 4	Within STZ only		STZ liraglutide versus STZ vehicle
Q9WVM8	Kynurenine/alpha‐aminoadipate aminotransferase	AADAT	Up
Q8CGC7	Bifunctional glutamate/proline–tRNA ligase	SYEP	Down
Q8CHT0	Delta‐1‐pyrroline‐5‐carboxylate dehydrogenase	ALDH4A1	Up
Set 5	Liraglutide reversed to healthy level		STZ vehcile versus healthy control and STZ liraglutide
P24270	Catalase	CATA	Down
P46412	Glutathione peroxidase 3	GPX3	Down
P97300	Neuroplastin	NPTN	Up

The proteins were divided into five different sets, depending on the significance patterns of the protein abundance characteristics. The direction of change in mean abundance level (up or down) is shown for each protein (see Table S5 for mean, SD and *P*‐values). ms, mass spectrometry.

**Figure 5:**
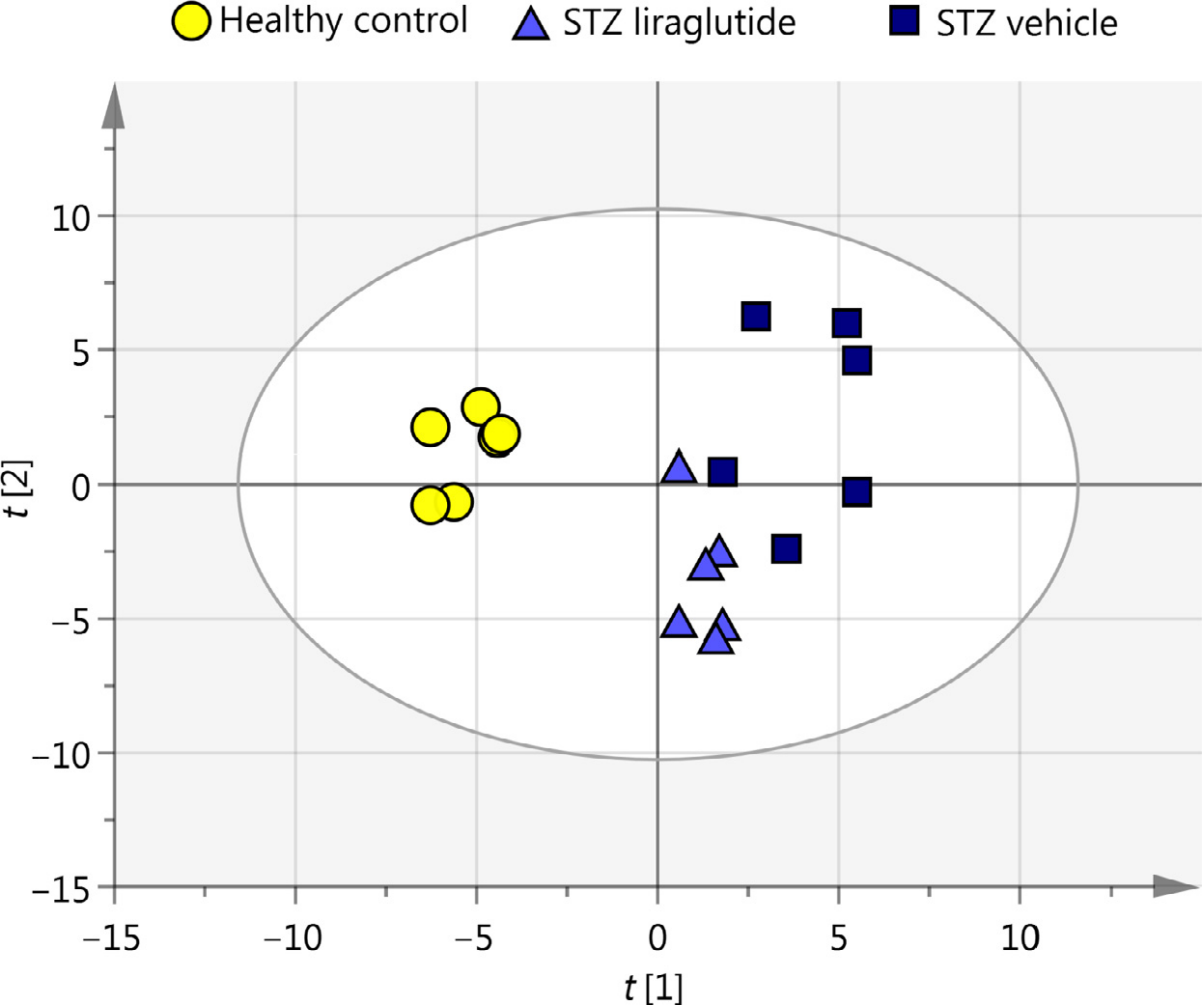
Unsupervised PCA of relative SRM intensities. The STZ liraglutide and STZ vehicle groups are separated in the PCA based on proteins with significant protein abundance levels from the normalized SRM data. The principal components explaining the separation are R2X[1]* *= 0.378 and R2X[2]* *= 0.296.

To check whether the proteins selected for SRM were sufficient to separate the STZ liraglutide from the STZ vehicle mice, the relative intensities of the proteins with significant protein expression in the SRM analysis were analyzed by PCA (Figure 5). In the targeted SRM‐based PCA, the STZ liraglutide group was separated from the STZ vehicle group without overlapping samples between the groups. This was not the case with the PCA plot of the complete shotgun data, where samples from the two STZ mouse groups were overlapping (Figure 3A).

## Discussion

It has previously been shown that liraglutide mediates renoprotective properties in STZ induced diabetic rats by specific effects involving NF‐*κ*B pathway (Zhou et al. 2014) and NAD(P)H oxidase inhibition (Hendarto et al. 2012). We investigated the overall effect of GLP‐1R agonism on the kidneys of STZ‐induced diabetic mice using liraglutide and a proteomics‐based approach. Distinct effects of liraglutide on the abundance levels were seen on several proteins, which led to separable protein profiles of the STZ vehicle and STZ liraglutide mice using supervised multivariate analysis (Figure 3B). Changes in protein abundance were seen in proteins with involvement in structural mechanisms like cell adhesion or cytoskeleton regulation. In addition, several of the proteins with insignificant protein abundance changes, possibly because of low power, were identified to be of importance for the group separation in the multivariate analyses. In NPTN and BRK1, the protein level was increased in the STZ vehicle compared to the healthy control mice and for NPTN, liraglutide restored or changed the protein level toward the levels in the healthy control mice (Figures 4A–B). NPTN is involved in cell adhesion and potentially neurite outgrowth (Berezin 2010) and BRK1 function involves cytoskeleton regulation and high levels have been reported to be associated with cell migration and proliferation in relation to cancer (Escobar et al. 2010). However, NPTN and BRK1 have not previously been described in association with diabetic kidney disease. The increased protein expression of proliferative and structural regulators in the STZ vehicle mice could indicate involvement in basement membrane thickening and ECM expansion in the glomeruli and the tubules, known to be early signs of kidney damage (Osterby 1972; Brito et al. 1998; Tervaert et al. 2010b). Our results suggest that liraglutide influences the abundances of some of these morphologically involved proteins, partly reversing the effect of the STZ‐treatment on the protein level in the STZ mouse model. The increased AER in the STZ mice, which is an early sign of kidney disease, was not altered by the addition of liraglutide, hence, the decreased prevalence of macroalbuminuria seen in the LEADER clinical trial in type 2 DM was not observed here (Mann et al. 2017).

A reduction in the protein level of PODXL was detected in the STZ liraglutide compared to the healthy control mice. Podocalyxin is a major constituent of the podocyte glycocalyx (Kerjaschki et al. 1984, 1986). The cause of the lower level in this study is not known.

Lower levels of the antioxidant defence protein GPX3 have been reported in the glomeruli of STZ rats compared to healthy control rats (Kim et al. 2014), which is in agreement with our findings, where liraglutide significantly increased the level of GPX3 compared to the STZ vehicle group (Figure 4D). GPX3 is mainly produced in the kidney proximal tubules (Avissar et al. 1994) and is the only GPX secreted to the bloodstream (Takahashi et al. 1987) where it mediates a reduction of the amount of hydrogen peroxide and lipid hydroperoxide (Ursini et al. 1985; Yamamoto and Takahashi 1993; Takebe et al. 2002). In an experimental study of inflammatory bowel disease in mice, GPX3 levels and activity in plasma were increased with over 60% compared to the healthy control group, concomitant with an increase in kidney but not intestinal GPX3 mRNA levels (Tham et al. 2002), showing the influence of renal GPX3. Besides the effect on GPX3, we found that liraglutide also influenced numerous ROS detoxification enzymes, increasing their abundances, here among several glutathione‐S‐transferases (seen in the multivariate analyses) and CATA (Figure 4E). The decreased CATA and GPX3 protein levels seen in the STZ vehicle mice were changed by liraglutide, restoring the abundance levels to those seen in the healthy control mice. Our data indicate that GLP‐1R agonism has a capacity of increasing proteins involved in antioxidant defence mechanisms in STZ‐treated mice. The data obtained will need further confirmation in an independent cohort.

The STZ‐induced diabetic mice did not gain as much weight as their healthy littermates and the weight gain was further decreased in the STZ liraglutide group. The difference in weight gain in the lean mouse model used in this study could have an effect on protein abundances in addition to the treatment with STZ and liraglutide. In type 1 DM clinical trials with liraglutide, only obese or over weight patients received liraglutide (Kuhadiya et al. 2013b, 2016; Dandona et al. 2018).

## Conclusion

In summary, we showed that GLP‐1R agonism influences the abundances of proteins most likely involved in ROS detoxification and of proteins with a possible connection to cell differentiation in the STZ mouse model. These changes could be mediated by a direct effect of liraglutide on the GLP‐1R in the kidney, by an indirect effect through a systemic response to GLP‐1R agonism or a combination of both. The indirect effects could be mediated by a lowered HbA_1c_ or by the reduced weight gain seen in the STZ liraglutide mice.

## Limitations of the Study and Future Perspectives

This study was initiated to investigate whether GLP‐1R agonism had an effect on protein abundances in early kidney damage in the STZ mouse model, a mouse model often used to mimic type 1 DM. The incretin response should not *per se* be absent in the healthy mice not receiving STZ intervention. Therefore, no effect of liraglutide was expected in a healthy liraglutide control group and such a control group was not included in the study. However, a healthy control group receiving liraglutide could have helped clarifying several of the questions arising from this study. Liraglutide induces weight loss, which it also did here. In this study we cannot separate the effects the reduced weight gain had on protein abundances compared to other mechanisms. Furthermore, to better mimic disease conditions, it would be interesting to investigate the effects of liraglutide supplemented with insulin in the STZ‐treated mice in a future study. To validate the location of the identified proteins, immunohistochemistry should be performed in a future study.

## Availability of Data and Materials

The shotgun MS data have been deposited to the ProteomeXchange Consortium (Vizcaíno et al. 2014) via the PRIDE partner repository (Vizcaíno et al. 2013) with the dataset identifier PXD003007 and 10.6019/PXD003007. The SRM MS data is available through PASSEL (Farrah et al. 2012) via Peptide Atlas with the dataset identifier PASS00839. A conversion table of ProteomeXchange accession numbers and the corresponding sample is shown in Table S7.

## Conflict of Interests

Novo Nordisk markets liraglutide for the treatment of diabetes and obesity. MHP and JNM are full‐time employees of Novo Nordisk and hold minor share portions as part of their employment. LL is a former employee of Novo Nordisk and holds minor share portions.

## Supporting information




**Table S1.** HbA1c (end‐point), blood glucose (BG), and raw peptide data from Progenesis software including peptide information.
**Table S2.** Transition list for SRM. RT, retention time.
**Table S3.** Unchanged protein abundances.
**Table S4.** SIMCA statistics.
**Table S5.** Protein with significant levels in the targeted MS analysis.
**Table S6.** Peptides used in the protein identification
**Table S7.** Guide to Proteome Exchange sample names.
**Figure S1.** (A) Albumin excretion rate, AER. (B) Kidney weight, right kidney. (C) Kidney weight per body weight. Kidney weight/body weight was higher in the two STZ mouse groups most likely because their body weights were decreased following STZ treatment compared to the healthy control group. The data is shown with mean and SD and statistical analyses were calculated using one‐way ANOVA with Tukey post hoc test for multiple comparisons. *P*<0.05 is considered significant.
